# Supplementary material for: Correlation of p53 oligomeric status and its subcellular localization in the presence of the AML-associated NPM mutant
Source: PLoS One. 2025 May 7;20(5):e0322096. doi: 10.1371/journal.pone.0322096 (PMC12058200; doi:10.1371/journal.pone.0322096)
Supplement: S1 Table — B) Primers used for construction of plasmids. (DOCX) [file pone.0322096.s001.docx]

**S1 Table**

**A**

| **Construct** | Abbreviation | **Construct** | Abbreviation |
| --- | --- | --- | --- |
| Cerulean_NPMmut | C_NPMmut | mVenus -p53-A353S | V_A353S |
| Cerulean_NPMwt | C_NPMwt | mVenus -p53-D352G | V_D352G |
| Cerulean_Δ117NPMmut | C_Δ117NPMmut | mVenus -p53-R337G | V_R337G |
| nowGFP-p53WT | G_p53wt | mRFP1-p53WT | R_p53wt |
| nowGFP-p53-L344A | G_L344A | mRFP1-p53-L344A | R_L344A |
| nowGFP-p53-L344P | G_L344P | mRFP1-p53-L344P | R_L344P |
| nowGFP-p53-A353S | G_A353S | mRFP1-p53-A353S | R_A353S |
| nowGFP-p53-D352G | G_D352G | mRFP1-p53-D352G | R_D352G |
| nowGFP-p53-R337G | G_R337G | mRFP1-p53-R337G | R_R337G |
| nowGFP-p53-I332S | G_I332S | mRFP1-p53WT-AAA | R_p53wt-ΔNLS |
| nowGFP-p53-R337D | G_R337D | mRFP1-p53-L344A-AAA | R_L344A-ΔNLS |
| nowGFP-p53-D352R | G_D352R | mRFP1-p53-L344P-AAA | R_L344P-ΔNLS |
| nowGFP-p53-R337D D352R | G_R337D-D352R | mRFP1-p53-A353S-AAA | R_A353S-ΔNLS |
| nowGFP-p53-D352R R337D | G_D352R-R337D | mRFP1-p53-D352G-AAA | R_D352G-ΔNLS |
| mVenus-p53WT | V_p53wt | mRFP1-p53-R337G-AAA | R_R337G-ΔNLS |
| mVenus-p53-L344A | V_L344A | mVenus-NPMmut | V_NPMmut |
| mVenus-p53-L344P | V_L344P |  |  |

**B**

**S1 Table:** A) List of constructs. B) Primers used for construction of plasmids.
