# Supplementary figures and images for: Correlation of p53 oligomeric status and its subcellular localization in the presence of the AML-associated NPM mutant
Source: PLoS One. 2025 May 7;20(5):e0322096. doi: 10.1371/journal.pone.0322096 (PMC12058200; doi:10.1371/journal.pone.0322096)

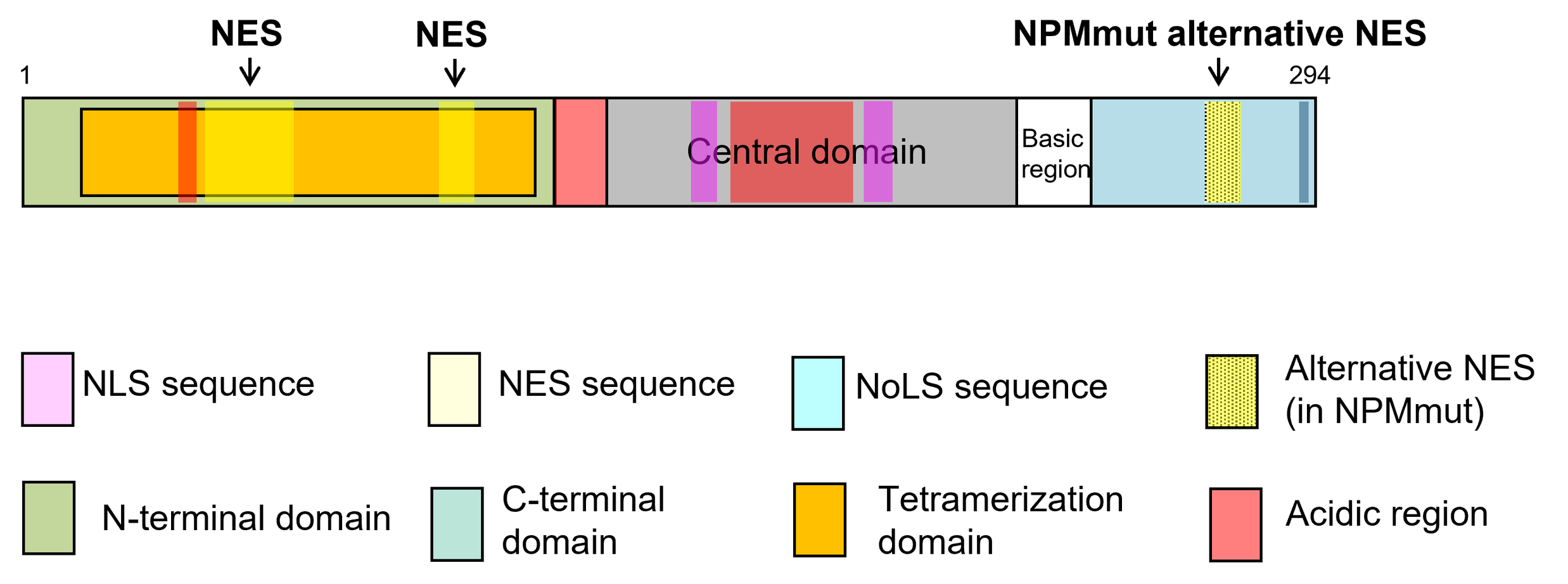

Supplement: S1 Fig — The alternative NES formed by the AML-related mutation is included in the scheme. (TIF) [file pone.0322096.s003.tif]

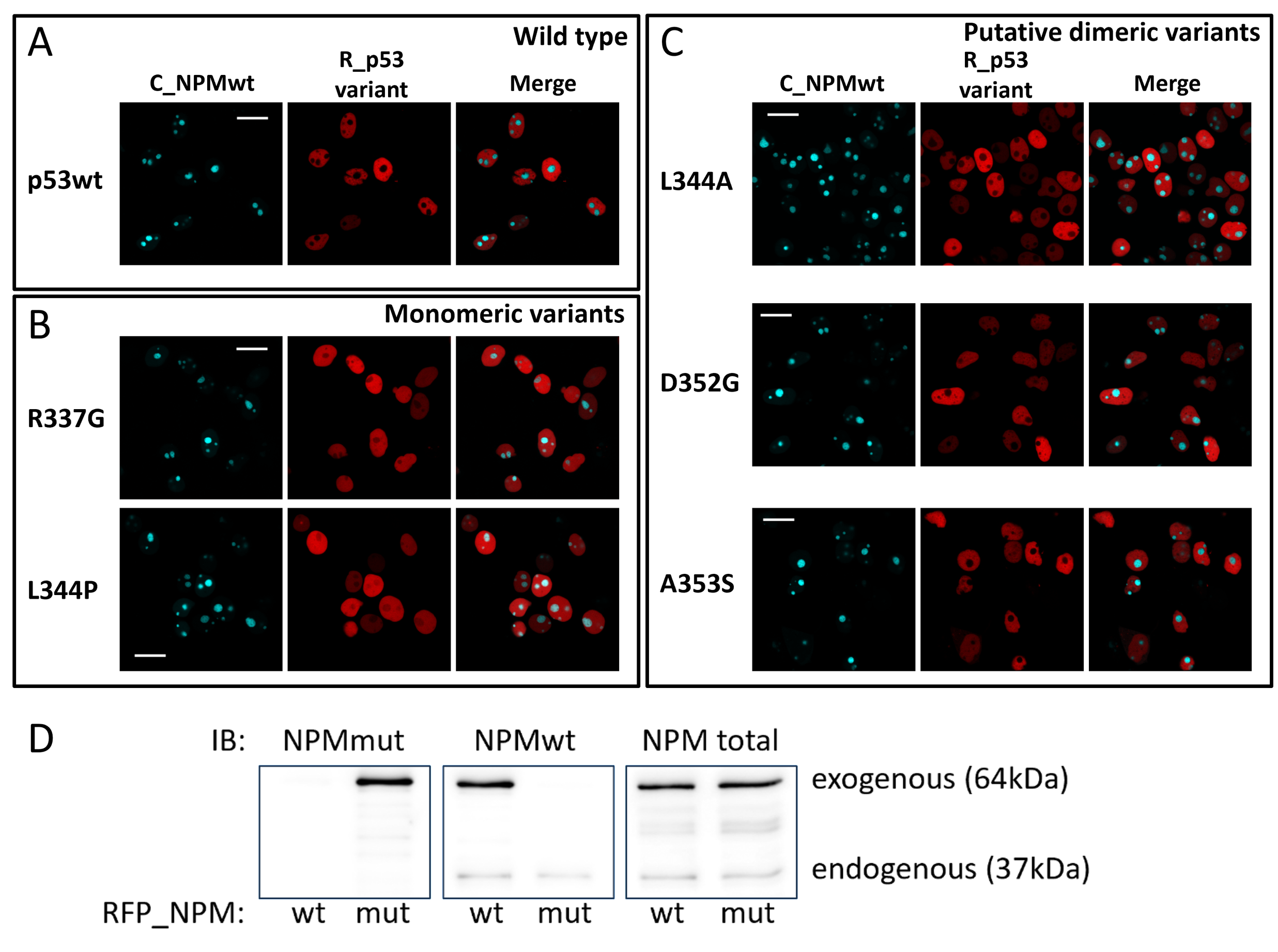

Supplement: S2 Fig — A) p53wt, B) monomeric variants, and C) putative dimeric variants. Bar represents 20 µm. (D) Expression of mRFP1-labeled NPM variants (wt or mut) in transfected HEK-293T cells. (TIF) [file pone.0322096.s004.tif]

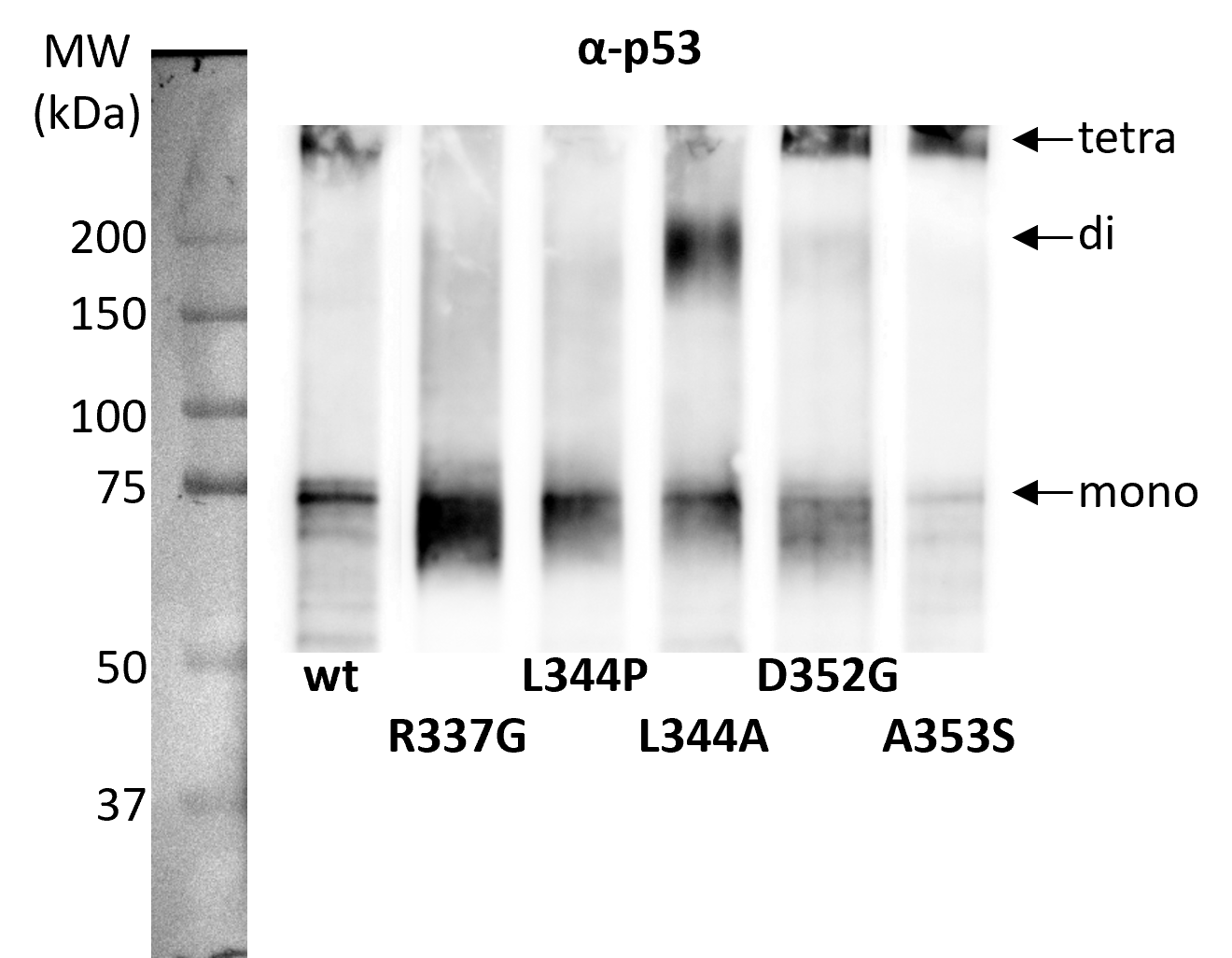

Supplement: S3 Fig — (TIF) [file pone.0322096.s005.tif]

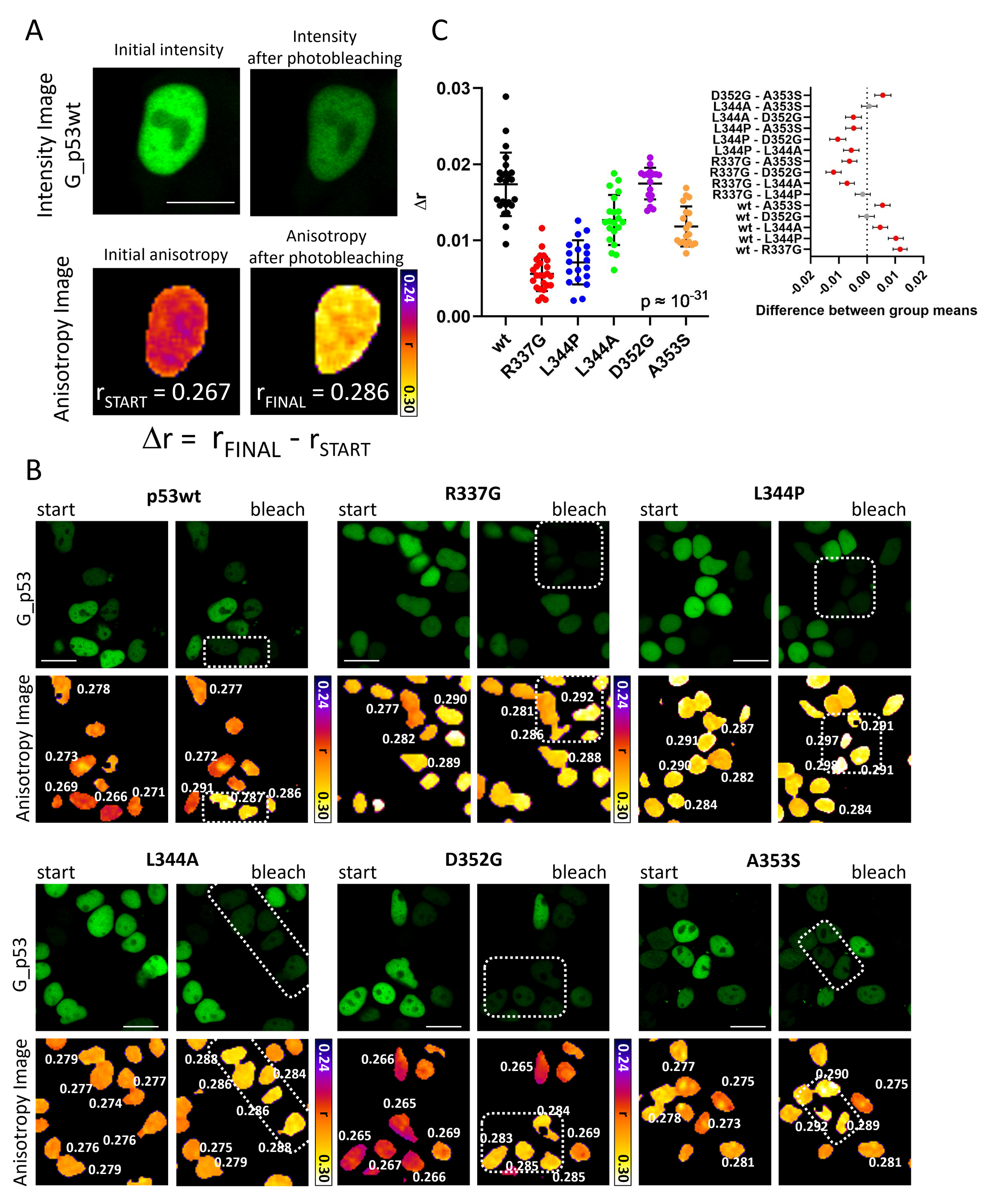

Supplement: S4 Fig — A) Demonstration of the homoFRET effect on nuclei of HEK-293T cells. The presence of the energy transfer manifests itself as an anisotropy increase in the photobleached sample, bar represents 10 μm. B) Representative anisotropy measurements in nowGFP-labeled p53 variants. Dotted line marks bleached cells with emission reduced to the 30% of the initial intensity. Bar represents 20 μm. C) Statistical evaluation of the anisotropy experiments (2–7 cells per image in 2–5 independent measurements). Mean values are plotted with ±SD (left), red symbols in the 95% confidence graph (right) mark significant differences between the appropriate pairs of samples (p < 0.05). (TIF) [file pone.0322096.s006.tif]

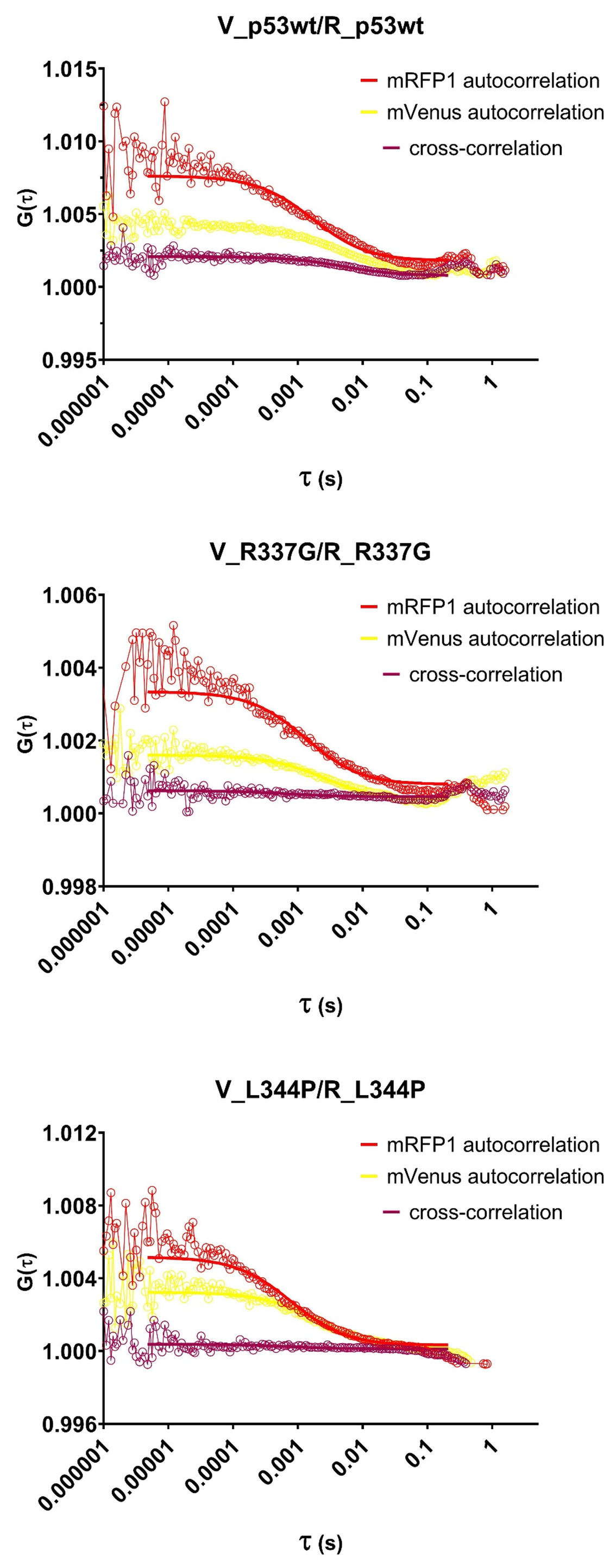

Supplement: S5 Fig — Solid lines are data fits. (TIF) [file pone.0322096.s007.tif]

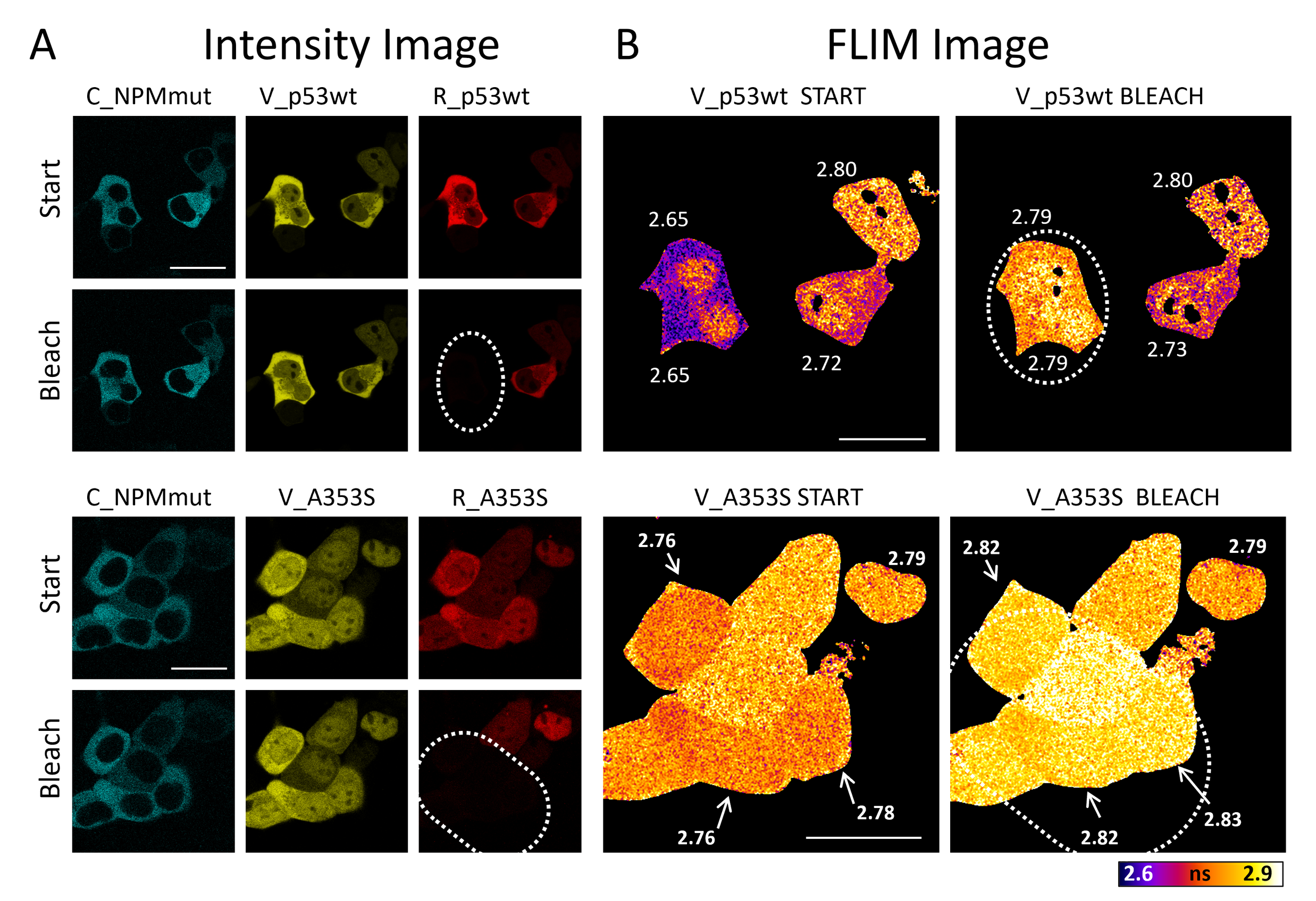

Supplement: S6 Fig — A) Intensity images of C_NPMmut, V_p53 and R_p53 before (START) and after (BLEACH) the photodestruction of mRFP1 by intense 561 nm irradiation. B) FLIM image before (left column) and after (right column) photodestruction of mRFP1 in cells marked with the dotted line. Numbers indicate lifetime in the cytoplasm. Bar represents 20 µm. (TIF) [file pone.0322096.s008.tif]

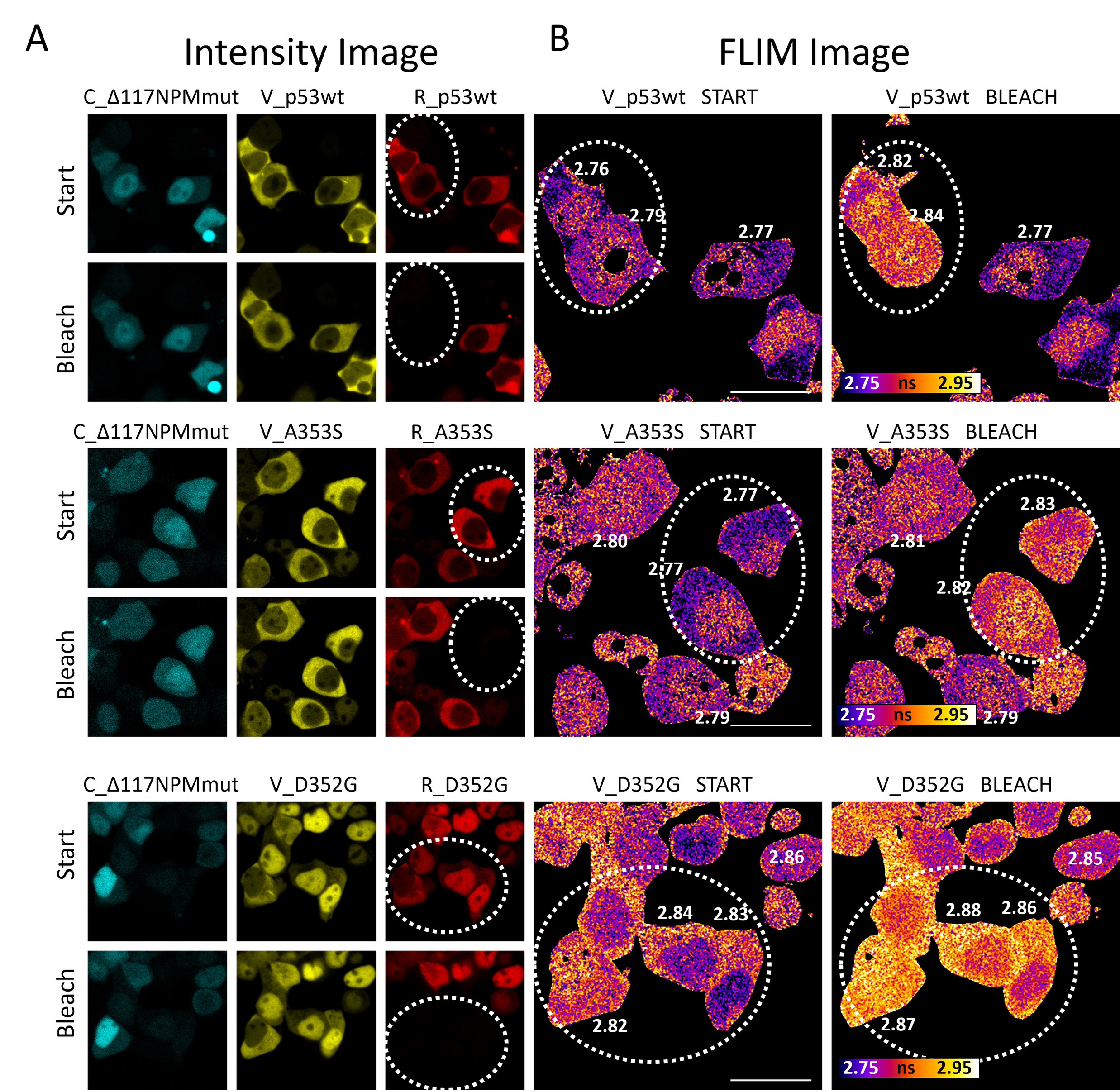

Supplement: S7 Fig — A) Subcellular localization of C_Δ117NPMmut, V_p53wt, and R_p53wt (1st, 2nd and 3rd column, respectively) before (START) and after (BLEACH) photodestruction of mRFP1 acceptor by 561 nm light. B) FLIM image before (START) and after (BLEACH) the mRFP1 photobleaching in cells bordered by the dashed line. Numbers refer to the emission lifetime in the cytoplasm. Bar represents 20 µm. (TIF) [file pone.0322096.s009.tif]
