# Supplementary material for: Correlation of p53 oligomeric status and its subcellular localization in the presence of the AML-associated NPM mutant
Source: PLoS One. 2025 May 7;20(5):e0322096. doi: 10.1371/journal.pone.0322096 (PMC12058200; doi:10.1371/journal.pone.0322096)
Supplement: Attachment — Submitted filename: Response to reviewers.docx [file pone.0322096.s012.docx]

**Reviewer #1:** The work represents a thorough analysis of the extend to which different p53 mutant, in particular those involved in oligomerisation are affected by the leukaemic NPCmut mutant.

The work is solid and well analysed but could do with some additional information as per my comments below.

1. What is the impact of the mutants - especially the set mutant on ability to interact/oligomerise with p73 or p63?

Thank you for this important and inspiring point. Unfortunately, in order to keep this work reasonably concise and well focused, we have not tested additional proteins from a family of p53 homologs. This suggested important aspect might be a subject of another separate study.

2. the oligmeric status of NPM is not well described - a similar diagram for NPM domain structure would be helpful and a little more introduction and discussion on this.

Diagrams of the NPM structure can be found in many publications, e.g. in Holoubek et al 2021 or Arregi et al 2015. We included such a diagram, based on Sasinkova 2022, in Fig. S1. In addition, essential NPM domains are specified on p.8 of the revised manuscript.

3. The authors state their own previous evidence on NPMmut being monomeric and cytoplasmic but NPM oligmerisation is regulated by AKT phosphorylation - this should be discussed. This would also allow them to extrapolate to other cancers where MAPK/AKT signalling is active and the impact of their work more broadly applicable across cancer where p53 mutants are found.

Thank you for this remark, which could also open a new research direction. Although interesting, one should be very cautious to generalize and extrapolate our AML-specific findings for other cancers, as the NPMmut occurs almost exclusively in AML. Nevertheless, we never stated that NPMmut is monomeric in the cytoplasm. The opposite is true. We have previously proved that NPMmut oligomerizes in the cytoplasm (Sasinkova 2021). This is now explicitly stated on the p.8 of the revised manuscript. Only the truncated NPMmut molecule marked here as Δ117NPMmut exhibits monomeric behaviour as described in (Sasinkova 2021). We have shown in Fig. S7 that the mislocalization pattern of our p53 variants in the presence of this monomeric NPM mutant is not significantly affected. We therefore concluded that it seems that the oligomeric status of NPMmut is not critical for the relocalization of p53 variants (Discussion, p.30 of the marked manuscript.). We also slightly reworded the abstract to avoid a potential confusion.

Specific inhibition of NPM oligomerization by AKT could be an interesting subject of another study. AKT was reported to inhibit NPM oligomerization due to its phosphorylation at Ser48 (Mitrea 2014), which had further consequence in p53wt stabilization and tumor suppression, and also in accumulation of specific p53 mutant (in-frame deletion of Thr126, Hamilton 2014). It confirms the important role of NPM oligomerization in tumorigenesis. However, several other NPM phosphorylation sites (e.g. Ser4, Thr95, Ser125 or Thr199) are regulated by other kinases, in particular by CKII and cdk1, and these modifications are also essential for the cell proliferation and apoptosis. Similarly, as can be seen from our set of p53 mutants, individual p53 mutations can manifest themselves differently.
**Reviewer #2:** In this manuscript Holoubek et al initiated a study to determine the ability of mutated p53 to oligomerize and translocate into the nucleus and or cytoplasm. Mutations tested include monomeric R337G and L344P, dimeric L344A, and multimeric D352G and A353S mutations. These mutations were tested in the context of a NPMmut , which is a mutant of NPM1 that is found in AML and results in the cytoplasmic localization of p53 (through the NPM-p53 interaction) rendering it a LOF. These studies focus on try to understand how NPMmut could pull out p53. This study is important and therefore publishable provided a few issues are resolved.

Major issues
Figure 2 is not properly controlled. In order to determine the effects of NPMmut on p53 localization a wt NPM would need to be also tested.

NPMwt is always present in AML cells expressing NPMmut (Falini et al 2007). The same is true for cells transfected with exogenous NPMmut. We have previously shown mainly nuclear p53wt localization in the presence of endogenous and exogenous NPMwt (Holoubek et al 2021), which contrasts with the cytoplasmic localization of p53wt in the presence of NPMmut. For convenience of readers, we have included in the supplemental material the localization figure of p53 variants in the presence of NPMwt (Figure S2).

Western blot is needed to ensure that the 2 cell lines expressing NPMmut and NPM wt are at the same expression levels.

The expression levels of NPMwt and NPMmut are comparable, as shown e.g. in (Brodska et al 2017, Sasinkova et al 2018, Holoubek et al 2021). Nonetheless, we included WB documenting comparable expression levels of the NPM variants in the supplemental Figure S2.

Missing from the study are in vitro interaction studies between the mutant p53 proteins and NPMmut, - as well as the NPM WT protein. Studies in Figure 8 use NPMmut but not the wt NPM. Is there a correlation between defects in localization to cytoplasm and the NPMmut (but not wt NPM) and p53 mutation status?

This paper is focused mainly on the mislocalization of p53 variants in the presence of NPMmut. As shown in the new Fig. S2, this mislocalization never happens in the presence of NPMwt for none of the p53 variants. To illustrate identical affinity of p53 variants to both NPMwt and NPMmut, we included representative WB image from immunoprecipitation of samples cotransfected with NPMwt and p53 variants to the Fig. 8. We are aware that the interaction of p53 with NPMwt could under physiological conditions shift, to some extent, the equilibrium and potentially facilitate partial nucleolar retention of p53. Nevertheless, in the transfected cells used in this manuscript the relative level of endogenous NPMwt is too low to bias the reported results and conclusions. The same holds for a small fraction of the mixed NPMmut/NPMwt oligomers in the cytoplasm. Those are the reasons why the interaction strength between p53 variants and NPMwt was not studied in detail in this paper.

Ttests are used throughout. Tables are shown which describe the results of many test per experiment. This is an incorrect stastical analysis. AMOVA analysis need to be done. A stastician needs to be consulted for the analysis of these figures and added to the authors of the paper.

According to the reviewer´s requirement, the ANOVA analysis with multiple comparison post-analysis was done in the revised manuscript. Overall ANOVA p-values were added to the original graphs and tables with individual p-values were replaced by 95% confidence value graphs. Numeric p-values comparing individual sample pairs were shifted to the supplement as Table S2. The description of the statistical analysis was updated in the Material and Methods section and noted in figure legends.

Minor Issues
FILM is not defined in the abstract

The meaning of the FLIM abbreviation was added to the abstract.

Figure 1 is confusing. The magnified region is ambiguous regarding its position relative to the full length protein. Is the magnified region within the hash marks? Or is it starting at the end of the grey portion, and ending with the C-terminal domain. Can the amino acid numbers be added to the beginning and end of the cutout region.

Fig 1 was reworked to better illustrate the p53 regions.
